# Neutrophil in the Pancreatic Tumor Microenvironment

**DOI:** 10.3390/biom11081170

**Published:** 2021-08-07

**Authors:** Lin Jin, Hong Sun Kim, Jiaqi Shi

**Affiliations:** Department of Pathology & Clinical Labs, Rogel Cancer Center and Center for RNA Biomedicine, University of Michigan, Ann Arbor, MI 48109, USA; linjin@med.umich.edu (L.J.); sunykim@umich.edu (H.S.K.)

**Keywords:** pancreatic ductal adenocarcinoma, tumor microenvironment, immune cells, neutrophil extracellular traps

## Abstract

Pancreatic ductal adenocarcinoma (PDAC) is a malignancy with a poor prognosis and low survival rates. PDAC is characterized by a fibroinflammatory tumor microenvironment enriched by abundant fibroblasts and a variety of immune cells, contributing to its aggressiveness. Neutrophils are essential infiltrating immune cells in the PDAC microenvironment. Recent studies have identified several cellular mechanisms by which neutrophils are recruited to tumor lesion and promote tumorigenesis. This review summarizes the current understanding of the interplay between neutrophils, tumor cells, and other components in the PDAC tumor microenvironment. The prognosis and therapeutic implications of neutrophils in PDAC are also discussed.

## 1. Introduction

Pancreatic ductal adenocarcinoma (PDAC) is one of the most lethal cancers with a five-year survival rate of 10% and is predicted to be the second leading cause of cancer-related death by 2030 [1,2]. The poor prognosis is associated with delayed diagnosis and the failure to achieve a durable response to available treatment modalities. Recent studies have highlighted the importance of the tumor microenvironment in PDAC progression and immune evasion. The dense desmoplastic stroma in PDAC contains abundant cancer-associated fibroblasts (CAFs). Another critical feature of the PDAC immune environment is the relative void of cytotoxic lymphocytes (CTLs) in the tumor core [3]. In order to overcome the tumor escaping host immune surveillance, it is crucial to understand the interaction between tumor cells and their surroundings.

Neutrophils are the most abundant immune cells in circulation and form an essential part of the innate immune system to respond against infection and inflammatory insults. Recent studies discovered an early homogeneous neutrophil progenitor subset in human bone marrow defined by surface markers CD71 and CD117 [4]. These CD71^+^ neutrophils proliferate and expand in the blood and tumors from cancer patients. It has become increasingly clear that neutrophils make up a substantial proportion of the immune infiltrate in a wide variety of cancers, including PDAC [5], breast cancer [6], colorectal cancer [7], melanoma [8], renal cell carcinoma [9], hepatocellular carcinoma [10], and others [11,12,13]. Intratumor neutrophils and high IL-8 levels are associated with poor outcomes to immune checkpoint inhibitors and worse survival in patients with advanced cancers. However, the role of neutrophils in tumor development is still unclear. Some studies have proposed classifying tumor-associated neutrophils (TANs) into two polarization states, tumor-suppressing N1 neutrophils and tumor-promoting N2 neutrophils [14]. In the lung cancer model, TGF-β inhibition leads to the recruitment and activation of N1-type neutrophils, which can kill tumor cells and inhibit tumor development [14]. Interferon-β signaling has been shown to polarize neutrophils to N1 phenotype [15]. Additionally, pro-inflammatory or immunostimulatory cytokines, such as interleukin-12 (IL-12), CXCL9, CXCL10, and CCL3, are released from N1 neutrophils and facilitate recruitment and activation of CD8^+^ T cells [16]. On the other hand, exposure to TGF-β transforms neutrophils to the N2 phenotype [14]. N2 neutrophils have been reported to have strong immunosuppressive and tumor-promoting functions, including the promotion of tumor metastases and angiogenesis [17,18]. N1 neutrophils are characterized as CD101^+^, CD177^+^, CD170^low^, CD54^+^, HLA-DR^+^, CD86^+^, and CD15^high^, whereas N2 neutrophils are CD170^high^, CD117^+^, Lectin-like oxidized LDL receptor 1 (LOX1)^+^, CD84^+^, junctional adhesion molecule-like (JAML)^+^, and PD-L1^+^ [15]. However, this dichotomized classification of TANs may be overly simplified because, in some tumors, such as 3-MCA-induced primary sarcomas, TANs have a mixed phenotype between N1 and N2 [14,15]. Furthermore, neutrophils can be trained to reprogram their transcriptome and epigenetics and take on the anti-tumor effect [19]. In the context of PDAC, TANs are associated with poor prognoses [20]. 

One way the tumor evades the host immune system is through a group of heterogeneous immature myeloid cells, called myeloid-derived suppressor cells (MDSCs). MDSCs suppress immune responses by multiple signaling pathways. MDSCs can induce antigen-specific CD8^+^ T cell tolerance via generating reactive oxygen species (ROS) and peroxynitrite [21]. In addition, MDSCs suppress natural killer (NK) cell cytotoxicity by inhibiting the activation of Stat5 [22]. Markers for MDSCs include CD33 and CD11b in humans and Gr-1 and Cd11b in mice [23]. Based on phenotypic features, MDSCs can be characterized into two major types, monocytic MDSCs (M-MDSCs) and granulocytic MDSCs (G-MDSCs) [24]. Mouse M-MDSCs are Ly6G^−^Ly6 C^high^, and G-MDSCs are Ly6G^+^ Ly6 C^low^ [25]. In humans, CD66b and CD15 are markers for M-MDSCs and G-MDSCs [25]. However, it is believed that the MDSCs described in most studies are, in fact, a subset of neutrophils [26,27] due to a significant overlap in the expression of functional molecules or surface molecules between neutrophils and G-MDSC [28].

In this review, we will focus on recent discoveries in the interaction between tumor cells, neutrophils, and other stromal cells in the TME of PDAC. We will also discuss the clinical implications of neutrophils in PDAC.

## 2. Interactions between Neutrophil and Tumor 

### 2.1. Tumor Cells Attract Neutrophils

**Figure 1:**
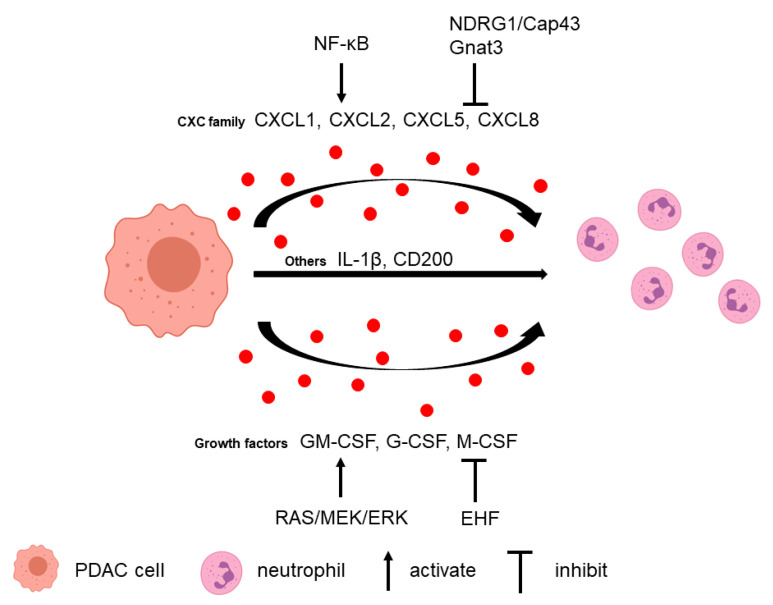
Tumor cell-secreted factors attract neutrophils. Tumor cells can release several factors to recruit neutrophils, such as the CXC family (CXCL1, CXCL2, CXCL5, and CXCL8), growth factors (GM-CSF, G-CSF, and M-CSF), IL-1β, and CD200. Different pathways regulate these factor secretions.

Neutrophils can be recruited to the PDAC microenvironment via multiple tumor-secreted factors (Figure 1). Several CXC families of chemokines have been attributed a major role in neutrophil recruitment, including CXCL1, CXCL2, CXCL5, and CXCL8 [29,30,31]. Neutrophils express CXC receptors CXCR1 and CXCR2 that can respond to the tumor-derived CXC family chemokines. CXCR1 binds to chemokines CXCL6 and CXCL8, and CXCR2 binds to chemokines CXCL1–3 and CXCL5–8 [32]. CXCR2 expression was upregulated and associated with tumor size in PDAC [33]. In Cxcr2 knockout PKF (*LSL-Kras^G12D/+^*; *Tgfbr2^flox/flox^, Ptf1a-Cre*) mice, infiltration of myeloperoxidase^+^ (MPO^+^) neutrophils and CD11b^+^Ly6G^+^ MDSCs to tumor is decreased compared to control animals [34]. In a KPC (*LSL-Kras^G12D/+^*; *LSL-Trp53^R172H/+^*; *Pdx1-Cre*) mouse model, the secretion of Cxcl1, Cxcl2, and Cxcl5 from tumor cells is increased and related to high numbers of MPO^+^ neutrophils compared to the normal pancreas [29]. Another study demonstrated that CXCL5 has the greatest fold increase in human PDAC and correlated with both tumor-infiltrating CD15^+^ granulocytes and neutrophil elastase^+^ (NE^+^) granulocytes [30]. Although there is limited literature on the specific role of the CXCL5/CXCR2 axis in regulating G-MDSC in PDAC, G-MDSC was shown to be positively correlated with CXCL5 level in human renal cell cancer, and blockage of CXCR2 reduced tumor weight with increased effector T cell infiltration in a renal cell cancer mouse model [35]. In a mouse melanoma model, CXCL5 is the primary chemokine to attract G-MDSCs to the tumor, which induced epithelial-mesenchymal transition, tumor cell migration, and metastasis [36]. In another gastric cancer model, MDSCs migration was partially attenuated with CXCR2 inhibitor, which suggested that the CXCL5/CXCR2 axis might play a role in MDSCs’ recruitment [37]. Furthermore, CXCL5 can recruit MDSCs to the tumor via CXCR2 and promote breast cancer progression [38].

In tumor cells, neutrophil-recruiting chemokine secretions can be regulated by several signaling pathways. For example, N-myc downstream regulated gene 1 (NDRG1)/Cap43 downregulates the expression of CXCL1, CXCL5, CXCL8, and VEGF in tumor cells, leading to the suppression of neutrophil infiltration [31]. In addition, ablation of α-gustducin (Gnat3) in Kras^G12D^-expressing pancreas increases the levels of different chemokines, including CXCL1 and CXCL2, which lead to G-MDSC recruitment and altered MDSC gene expression in early neoplasia [39]. NF-ĸB is another critical signaling pathway in which these chemokines are regulated. Knockdown of Vasohibin-2 (VASH2), an endothelium-derived angiogenesis inhibitor, in murine pancreatic cancer cells downregulates NF-ĸB signaling, which results in decreased infiltration of CD11b^+^ Ly6G^+^ G-MDSC mediated by low Cxcl2 and Cxcl5 [40]. Another study found that in response to KRAS/MEK inhibition, NF-ĸB activation induces CXCL5 secretion in pancreatic cancer cells, which elevated CD11b^+^Ly6G^+^ neutrophil infiltration [41]. 

Growth factors secreted by tumor cells also play a role in neutrophil recruitment. Granulocyte macrophage colony-stimulating factor (GM-CSF), granulocyte colony-stimulating factor (G-CSF), and monocyte colony-stimulating factor (M-CSF) are upregulated in PDAC cells, promoting the survival and recruitment of G-MDSC and CD11b^+^ Ly6G^+^ neutrophils in vivo [42]. G-CSF expression is upregulated by activating the RAS/MEK/ERK pathway in PDAC cells through the ETS transcription factor [43]. In addition, ETS homologous factor (EHF) directly suppresses the GM-CSF level by binding to its promoters. EHF loss in tumor cells relieves the transcriptional suppression of GM-CSF, which induces MDSCs conversion, expansion, and function [44].

Other cytokines and proteins derived from pancreatic cancer cells also influence neutrophil infiltration. Tumor cell-derived IL-1β contributes to neutrophil infiltration since depletion of tumor cell-derived IL-1β significantly decreases the stromal accumulation of CD11b^+^ Gr1^+^ MDSC and CD11b^+^ Ly6G^+^ TANs [45]. CD200 (OX-2; OX-90) from the epithelial PDAC cells and α-SMA+ stromal cells enhances CD200R^+^ MDSC expansion and increases their immunosuppressive activity on T-cell proliferation [46]. Tumor cell-intrinsic ubiquitin-specific protease 22 (USP22) regulates immune cell infiltration in implanted PDAC tumors [47]. USP22 ablation results in decreased total myeloid cells and G-MDSCs. Reg3g can promote pancreatic carcinogenesis, and its overexpression in tumor cells enhances CD11b^+^Gr1^+^ MDSC recruitment [48].

### 2.2. Neutrophils Promote Tumor Cell Survival and Metastasis

Multiple studies have demonstrated that tumor-infiltrating neutrophils have a pro-tumor role in PDAC. PDACs can be subtyped into squamous, aberrantly differentiated endocrine exocrine, pancreatic progenitor, and immunogenic subsets based on transcriptomic data [49]. PDACs can also be classified based on whether they have normal or activated stroma [50]. A study used gene set variation analysis (GSVA) to compare the enrichment of these PDAC subset gene signatures between TAN-high and TAN-med/low PDACs [41]. TAN-high PDACs were found to significantly enrich genes in the squamous and normal stroma subtypes, which had the poorest prognosis. Several studies in PDAC have found neutrophil depletion could inhibit tumor growth and metastasis. Depleting G-MDSC in the KPC mouse model using anti-Ly6G antibody, 1A8, resulted in an increase in cleaved caspase-3 (CC3)-positive tumor cells, which suggested that depletion of G-MDSC increased tumor epithelial apoptosis [42]. Another study used anti-Ly6G antibody 1A8 in KPC mice, which reduced the number of MPO^+^ cells infiltrating tumors. Although there was no difference in overall or tumor-free survival, depletion of neutrophils abrogated tumor metastasis [29]. In KPC cell-derived orthotopic tumors, anti-Ly6G treatment delayed tumor growth, and a combination of anti-Ly6G and anti-PD-1 treatment showed a synergistic anti-tumor effect [51]. Importantly, PDAC patient-derived neutrophils significantly promoted the migration and invasion of pancreatic cancer cells in wound healing assay and transwell assay in vitro, whereas neutrophils derived from normal individuals did not [52]. 

## 3. Interactions between Neutrophil and TME

### 3.1. T Cell

**Figure 2:**
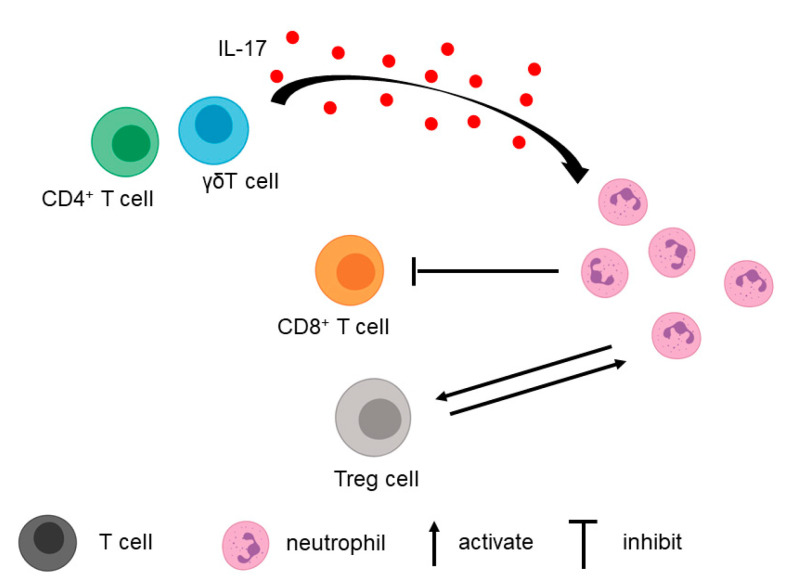
Interactions between neutrophils and T cells. IL-17 secreted from CD4^+^ T cells and γδT cells attracts neutrophils. Neutrophils can inhibit CD8^+^ T cell proliferation, activation, and recruitment. Neutrophils and Treg cells can activate each other.

In addition to tumor cells, T cells in the TME contribute to the recruitment of neutrophils (Figure 2). A study by Zhang et al. found that IL-17, a cytokine secreted primarily by CD4^+^ and γδT cells, played a role in neutrophil recruitment [51]. When the IL-17 signaling pathway was blocked using anti-IL17/anti-IL17R neutralizing antibodies in the orthotopic pancreatic cancer mouse model, myeloid cell recruitment decreased, whereas the total number of CD8^+^ T cells and activated CD8^+^ T cells increased [51,53]. This finding shows that neutrophils and T cells can influence each other.

Different subsets of T cells can interact with TANs. For example, MDSCs can directly interact with Treg [54]. When MDSCs are depleted using anti-Gr-1 RB6–8C5 antibody, Foxp3^+^ Treg recruitment is inhibited in PDAC [54]. Furthermore, MDSCs are able to induce Treg cell proliferation in a cell–cell-dependent manner. Treg cells, in turn, can affect the survival and/or the proliferation of MDSCs [54].

Organoid/immune cell co-culture models demonstrated that G-MDSCs could inhibit CTL proliferation. Depletion of G-MDSCs using cabozantinib, a tyrosine kinase inhibitor known to deplete MDSCs, increased CTL proliferation and effector function, resulting in cancer cells’ sensitization to anti-PD-1/PD-L1 treatment [55]. G-MDSC (CD11b^+^ Gr-1^high^ Ly6C^int^) and M-MDSC (CD11b^+^ Gr-1^int^ Ly6C^high^) isolated from KPC mice suppressed T cell proliferation and induced apoptosis of activated T cells in vitro. Depleting G-MDSC using anti-Ly6G antibody significantly increased the percentage and the absolute number of CD8^+^ T cells in KPC mouse tumors [42].

A different study found that MDSC isolated from bone marrow or KPC tumors inhibited proliferation and induced apoptosis of CD8^+^ T cells in the presence of dendritic cells (DC) presenting a high-affinity cognate peptide, even following T cell initial activation by DCs [56]. In addition, MDSCs could induce DNA damage and p53 pathway activation in CD8^+^ T cells through an iNOS-dependent pathway [56].

### 3.2. Fibroblasts, Macrophages, and Extracellular Matrix

Pancreatic stellate cells (PSCs) and CAFs are essential components in the pancreatic TME. These stromal cells also interact with immune cells, including neutrophils. One study demonstrated that PSCs induced MDSC expansion in the orthotopic PDAC model [57]. In the same study, co-transplantation of PSCs and tumor cells significantly increased G-MDSCs infiltration in the tumor [57]. In turn, neutrophil DNA could activate PSC and increase PSC proliferation, enhancing the production of the matrix metallopeptidase 2 (MMP2) and matrix metallopeptidase 9 (MMP9) secreted by PSCs through interaction with the receptor for advanced glycation end productions (RAGE) [58]. Another study showed infiltrated neutrophils in PDAC reprogramed the functional status of stellate cells, which included reduced collagen production and increased protease synthesis of PSCs [59]. 

Tumor-associated macrophages (TAMs) are also significant components of the microenvironment and have been demonstrated to be correlated with worse patient prognosis in PDAC [60]. In a preclinical model, targeting both neutrophils and macrophages enhanced chemotherapeutic efficacy and improved the survival of mice with orthotopic PDAC tumors [30]. In breast cancer, IL-10 from MDSCs reduced IL-12 production by macrophages, resulting in the polarization towards an M2 phenotype [61]. Further studies on the interaction between neutrophils and macrophages in PDAC would be beneficial.

Additionally, neutrophils can reprogram the extracellular matrix of PDAC. Targeted depletion of G-MDSC in the KPC mouse model decreases ECM deposition and the appearance of patent blood vessels, with increased vessel diameters [42]. Neutrophil-derived MMP9 has a direct and robust proangiogenic effect independent and additive to PDAC-derived VEGF [62].

## 4. Neutrophil Extracellular Trap in PDAC

The formation of neutrophil extracellular trap (NET) is a cellular function of neutrophils. Release of NET, called NETosis, is considered a defense mechanism to trap and kill bacteria and other pathogens. NETs consist of extracellular DNA and several proteins, including MPO and neutrophil elastase (NE) [63]. Studies across different cancers have suggested that NETs play an essential role in tumor progression and metastasis [64,65,66]. NETs induce the awakening of dormant cancer cells to form metastasis in lungs in mouse models of breast and prostate cancer [67]. The role of NETs in PDAC drew much attention as our understanding of neutrophils in tumors improved.

Many studies have demonstrated that pancreatic cancer cells induce NET formation. For example, the conditioned media from PDAC cell line AsPC-1 was able to induce NET formation, and NETs could promote AsPC-1 cell migration and invasion as well as angiogenesis in vitro [68]. Another group found that conditioned media from KPC cells previously exposed to IL-17 induced NETosis in mouse neutrophils in vitro [51]. Moreover, serum from PDAC patients significantly increased NET formation and reduced NET degradation [51]. Tumor-derived protein tissue inhibitor of metalloproteinases-1 (TIMP-1), which correlated with poor prognosis in PDAC, directly triggered the formation of NETs. This effect depended on the interaction of TIMP-1 with its receptor CD63 and subsequent ERK signaling [69]. In addition to tumor cells, CAF-conditioned media can also induce ROS-dependent NETosis by secreting Amyloid β A4 protein. In turn, NET formation promotes CAF expansion, contractility, and deposition of matrix components supportive of tumor growth. 

Recent studies suggested that NETs contribute to PDAC development. Electron microscope images of co-cultured pancreatic cancer cells with neutrophils showed that NETs could capture pancreatic cancer cells by their spider web-like structures [70]. The same study further demonstrated that NET-derived HMGB1, which could be degraded by thrombomodulin, potentiated tumor aggressiveness by inducing epithelial-mesenchymal transition (EMT) [70]. In another study, when NET supernatant was introduced, pancreatic cancer cell proliferation increased in a dose-dependent manner [52]. This increase in proliferation was reversed when DNase was present. Similarly, conditioned media from activated neutrophils promoted migration, invasion, and EMT in PDAC cells via the IL-1β/EGFR/ERK pathway [52]. Protein Arginine Deiminase 4 (PAD4), which citrullinates histones, is critical in the NET release [71]. PAD4 inhibitor, GSK484, treatment reduced NET formation and completely suppressed pancreatic tumor growth in the xenograft mouse model [72].

## 5. Clinical Implications of Neutrophil and NET in PDAC

### 5.1. Prognosis in PDAC

The neutrophil-to-lymphocyte ratio (NLR) offers critical prognostic information in PDAC as well as breast cancer, lung cancer, and other types of cancer [73,74]. Studies have demonstrated that increased NLR correlates with a poor prognosis in patients with resectable and unresectable pancreatic cancer [30,75,76,77]. The combination of NLR and platelet-lymphocyte ratio significantly improves the prognostic stratification of metastatic PDAC patients [78]. For PDAC patients undergoing chemotherapy, both baseline NLR and post-chemotherapy NLR change (the post-chemotherapy NLR divided by the baseline NLR) are independent prognostic factors in overall survival [79]. Relative changes of NLR after two doses of immune checkpoint blockade are significantly correlated with an increased risk of death for PDAC patients [80]. Increased CXCR2^+^ neutrophils in the blood and bone marrow are correlated with worse clinical outcomes [30]. Baseline neutrophilia and increased absolute neutrophil count are associated with worse overall survival in patients with locally advanced pancreatic cancer [81,82].

Intratumoral neutrophils are also correlated with PDAC patient outcomes. CD177, a marker for neutrophils, is negatively correlated with the overall survival of PDAC patients [83]. CD66b is another marker for neutrophils. Higher levels of CD66b^+^ tumor-infiltrating neutrophils are significantly associated with shorter survival in PDAC patients [5]. Another group found that a high CD15^+^ TAN to CD8^+^ lymphocyte ratio in PDAC was associated with worse overall survival [30].

A recent study showed that plasma NET levels using SYTOX-positive areas could also predict the survival of PDAC patients [69]. High plasma levels of NET markers were indicators of increased death risk of PDAC patients. The combination of plasma TIMP-1 and NETs markedly enhanced the prognostic power. TIMP-1^high^ NET^high^-patients had an almost sixteen-fold increase in mortality rate compared to TIMP-1^low^ NET^low^-patients. Notably, a combination of plasma levels of TIMP-1 and NETs with the clinically established marker CA19–9 exhibited a superior prognostic value as compared to CA19–9 alone. Tumor-Infiltrating CitH3-positive NETs were associated with poor overall survival and recurrence-free survival in PDAC patients. In addition, the combination of NETs with the 8th edition TNM staging system generated a novel model that improved the predictive accuracy for survival [84].

### 5.2. Neutrophil as a Potential Therapeutic Target in PDAC

The existing treatment of PDAC patients includes surgical resection, chemoradiation therapy, and immunotherapy, but only a small proportion of patients benefit from available therapies. Based on an increasing understanding of the role of TME in PDAC, neutrophils have emerged as a potential therapeutic target. Several preclinical studies with different mouse models have shown encouraging results of targeting neutrophils in pancreatic cancer (Table 1). The majority of neutrophil targeting preclinical studies use CXCR2 inhibitors or Ly6G antibodies [30]. This approach is in line with the main classes of current and past drug targets being receptors, among others [85]. In addition, the preference of CXCR2 as a target might lie in the fact that CXCR2 blockage not only inhibits the CXCL5/CXCR2 axis but also blocks the effect of other ligands of CXCR2, including CXCL1–3 and CXCL6–8. In breast cancer, CXCR2 inhibitors were demonstrated to be safe and tolerable, and relevant clinical trials are currently underway (NCT02370238) [86]. However, ligands, including cytokines, have become more important drug targets in the past two decades, largely due to the increased antibody-based therapies. Although no study was found in PDAC, CXCL5 inhibition emerged as a new strategy to attenuate tumor angiogenesis and thus slow tumor progression [87]. CXCL5 neutralizing antibodies treatment decreased the metastatic rate of breast cancer cells [88]. In lung cancer, CXCL5 neutralizing antibody improved the efficacy of tyrosine kinase inhibitor gefitinib without increased side effects [89]. More studies need to be conducted to investigate the therapeutic potential of CXCL5 blockage in PDAC.

## 6. Conclusions and Perspectives

More and more studies show that neutrophils play crucial roles in influencing the PDAC microenvironment and PDAC progression. The interactions among neutrophils, PDAC cells, and other components in TME are complex and are regulated by several signaling pathways. In addition, several parameters related to neutrophils are used as potential biomarkers for predicting clinical prognosis in PDAC patients. Moreover, unraveling the exact mechanisms of neutrophil function in PDAC development and new findings in preclinical and clinical studies will undoubtedly further our understanding of PDAC pathophysiology and provide a breakthrough in the treatment.

## Figures and Tables

**Table 1:** Preclinical studies of targeting neutrophils in pancreatic cancer.

Mouse Model	Cell Line	Treatment	Results	Reference
*LSL-Kras^G12D/+^; Tgfbr2^flox/flox^*; *Ptf1a-cre* (PKF) *Cxcr2^+/+^* and *Cxcr2^+/−^*	-	-	Cxcr2 knockout significantly extends the survival of PDAC mice.	[34]
*LSL-Kras^G12D/+^*; *LSL-Trp53^R172H/+^*; *Pdx1-Cre* (KPC) *Cxcr2^+/+^* and *Cxcr2^-/−^*	-	anti-Ly6G antibody 1A8 CXCR2 signaling inhibitor pepducin CXCR2 inhibitor AZ13381758	Cxcr2 deletion completely abrogated metastasis. Depletion of Ly6G^+^ cells resulted in strong suppression of metastasis. Pepducin treatment resulted in a significant increase in survival. CXCR2 inhibitor treatment prolonged the survival of KPC mice and significantly protected from metastasis.	[29]
KPC	-	anti-Ly6G antibody 1A8	Depletion of Ly6G^+^ cells induced tumor cell apoptosis.	[42]
C57BL/6	KPC cells	anti-Ly6G antibody	Depletion of Ly6G^+^ cells significantly delayed tumor growth in the orthotopic model.	[51]
C57BL/6	KPC cells	CXCR2 inhibitor SB225002 anti-Ly6G antibody 1A8	CXCR2 inhibitor or depletion of Ly6G^+^ cells reduced tumor burden in subcutaneous and orthotopic models.	[30]
*Cxcr2^+/+^* and *Cxcr2^−/−^*	KPC cells	-	CXCR2 ablation inhibited tumor growth in the subcutaneous model.	[41]
Padi4^+/+^ and Padi4^−/−^	Panc02 cells	-	NET-deficiency decreased pancreatic tumor burden and increased survival.	[58]
C57BL/6	KPC cells	PAD4 inhibitor GSK484	GSK484 treatment completely suppressed tumor growth in the subcutaneous model.	[72]
C57BL/6	KPC cells	anti-Ly6G antibody 1A8	Depletion of Ly6G+ cells reduced the formation of liver metastases in the intrasplenic model.	[90]
